# Absence of X-chromosome dosage compensation in the primordial germ cells of *Drosophila* embryos

**DOI:** 10.1038/s41598-021-84402-7

**Published:** 2021-03-01

**Authors:** Ryoma Ota, Makoto Hayashi, Shumpei Morita, Hiroki Miura, Satoru Kobayashi

**Affiliations:** 1 Department of Biosciences, Faculty of Science and Engineering, Teikyo University, Utsunomiya, Tochigi 320-8551 Japan; 2 Division of Integrated Science and Engineering, Graduate School of Science and Engineering, Teikyo University, Utsunomiya, Tochigi 320-8551 Japan; 3 Life Science Center for Survival Dynamics, Tsukuba Advanced Research Alliance (TARA), University of Tsukuba, Tsukuba, Ibaraki 305-8577 Japan; 4 Graduate School of Life and Environmental Sciences, University of Tsukuba, Tsukuba, Ibaraki 305-8577 Japan; 5 Present Address: Molecular Biology, Cell Biology and Biochemistry, Brown University, Providence, RI 02906 USA

**Keywords:** Cell biology, Developmental biology

## Abstract

Dosage compensation is a mechanism that equalizes sex chromosome gene expression between the sexes. In *Drosophila*, individuals with two X chromosomes (XX) become female, whereas males have one X chromosome (XY). In males, dosage compensation of the X chromosome in the soma is achieved by five proteins and two non-coding RNAs, which assemble into the male-specific lethal (MSL) complex to upregulate X-linked genes twofold. By contrast, it remains unclear whether dosage compensation occurs in the germline. To address this issue, we performed transcriptome analysis of male and female primordial germ cells (PGCs). We found that the expression levels of X-linked genes were approximately twofold higher in female PGCs than in male PGCs. Acetylation of lysine residue 16 on histone H4 (H4K16ac), which is catalyzed by the MSL complex, was undetectable in these cells. In male PGCs, hyperactivation of X-linked genes and H4K16ac were induced by overexpression of the essential components of the MSL complex, which were expressed at very low levels in PGCs. Together, these findings indicate that failure of MSL complex formation results in the absence of X-chromosome dosage compensation in male PGCs.

## Introduction

In some reproductive animals, sex chromosomes play key roles in sex determination and differentiation^1^. In such animals, sex-chromosome constitution differs between females and males. For example, in mammals and *Drosophila*, females and males carry two X chromosomes (XX) and one X chromosome (XY), respectively^1^. This difference in the chromosome constitution causes an imbalance in the number of X-linked genes between the sexes; however, their expression can be equalized between XX females and XY males through a mechanism called dosage compensation^2^.

In the somatic cells of *Drosophila* males, expression of X-linked genes is upregulated twofold by the dosage compensation mechanism^3^. This upregulation is mainly mediated by the male-specific lethal (MSL) complex. Exclusively in males, this complex binds the X chromosome to acetylate lysine residue 16 in histone H4 (H4K16ac), which in turn hyperactivates X-linked genes^4,5^. The MSL complex contains the essential components for catalysis, MSL-1 (a scaffold protein), MSL-2 (a ubiquitin ligase), MSL-3 (a chromodomain protein), MOF (a histone acetyltransferase), and MLE (a DNA/RNA helicase), in addition to the non-coding RNAs *roX1* and *roX2*^3^. In the soma, the transcript encoding MSL-2 is repressed translationally in females by *Sex-lethal* (*Sxl*), which is a binary switch gene that regulates sex determination and dosage compensation, allowing formation of the MSL complex only in males^6–8^.

In contrast to the soma, the existence of dosage compensation in the *Drosophila* germline remains controversial. Rastelli and Kuroda showed that in the germline cells of adult testes, MLE does not localize to the X chromosome, and MSL-3 is not expressed, although H4K16ac is present^9^. Furthermore, Gupta and colleagues performed a microarray analysis that revealed twofold upregulation of X-linked genes in the germline of adult testes^10^. However, recent transcriptome analyses do not support the idea that dosage compensation occurs in the germline of adult testis^11–13^. The discrepancies may result from differences in sample preparation or the gene sets selected for each analysis. Likewise, varying degrees of contamination of somatic cells in the germline samples could yield dissimilar conclusions. Furthermore, meiotic inactivation of X-linked genes in spermatocytes^14^ raises concerns about the validity of the conclusions.

In this study, we focused on primordial germ cells (PGCs) in embryos, as dosage compensation is linked with sex determination and is an early event in the soma that differs between the sexes^3,6,15,16^. In *Drosophila*, PGCs are formed in the posterior pole region of early embryos, and then migrate through the embryo (middle embryogenesis) to reach the embryonic gonads (late embryogenesis). In migrating PGCs, *Sxl* is expressed in a female-specific manner and induces female fate^17^, although its downstream targets in these cells are distinct from those in the soma^18–22^. By contrast, male PGCs within gonads initiate male fate in response to JAK/STAT signals from the gonadal soma^23,24^. These observations led us to investigate whether dosage compensation occurs in PGCs during these embryonic stages.

In this study, we found that dosage compensation was undetectable in PGCs during middle to late embryogenesis. Consistent with this, H4K16ac was undetectable in male PGCs. All transcripts encoding the components of the MSL complex, except for MOF, were expressed at very low levels in PGCs. Moreover, overexpression of *msl-1*, *msl-2*, *msl-3*, and *roX2* induced hyperactivation of X-linked genes and H4K16ac in male PGCs. Our observations strongly suggest that dosage compensation is absent in male PGCs, due to the failure of MSL expression.

## Materials and methods

### Flies

Flies were maintained on standard *Drosophila* medium at 25 °C. Strains *y w*, *vasa-EGFP*^25^, and *nos-Gal4-VP16* (*nos-Gal4*)^26^ are maintained in our lab; *UAS-RedStinger* (*UAS-RFP*, stock No. 8545)^27^, M{3xP3-RFP.attP'}ZH-2A (24480)^28^, and M{3xP3-RFP.attP'}ZH-58A (24484)^28^ were obtained from the Bloomington *Drosophila* Stock Center.

### Production of transgenic flies

To produce flies carrying *UAS-msl-1* and *UAS-msl-2*, total RNA was isolated from 4 to 8 h AEL (after egg laying) embryos using ISOGEN (NIPPON GENE, 311-02501), and cDNA was synthesized using SuperScript III (Thermo Fisher Scientific, 18080093). The open reading frames (ORFs) of *msl-1* and *msl-2* were amplified from the cDNA using primer pairs msl-1Fw/msl-1Rv and msl-2Fw/msl-2Rv, respectively (Table S1). The *msl-1* and *msl-2* ORFs were cloned into *Kpn*I-digested pUASp-K10-attB^29^ using the In-Fusion HD Cloning Kit (Takara, Z9648N). DNA spanning from the 5′ terminus of the GAGA-binding site to the 3′ terminus of K10, which contains the *msl-1* ORF, was amplified using primers pUASp-K10-attBFw/pUASp-K10-attBRv (Table S1), and the amplicon was cloned into *Psi*I-digested pUASp-K10-attB containing the *msl-2* ORF. The resultant vector was injected into M{3xP3-RFP.attP'}ZH-58A embryos to produce flies carrying *UAS-msl-1* and *UAS-msl-2* on the second chromosome. *w*^+^ transformants were collected and used to establish homozygous stocks.

To produce flies carrying *UAS-msl-3* and *UAS-roX2*, the *msl-3* ORF and full-length *roX2* (RD-type isoform) were amplified from cDNA obtained from 4 to 8 h AEL embryos using primers msl-3Fw/msl-3Rv and roX2Fw/roX2Rv (Table S1), respectively. The *msl-3* ORF and *roX2* fragment were cloned into *Kpn*I-digested pUASp-K10-attB using the In-Fusion HD Cloning Kit. DNA spanning from the 5´ terminus of the GAGA-binding site to the 3´ terminus of K10, which contains the *msl-3* ORF, was amplified using primers pUASp-K10-attBFw/pUASp-K10-attBRv (Table S1), and the amplicon was cloned into *Psi*I-digested pUASp-K10-attB containing the *roX2* fragment. The resultant vector was injected to M{3xP3-RFP.attP}ZH-2A embryos to produce flies carrying *UAS-msl-3* and *UAS-roX2* on the X chromosome. *w*^+^ transformants were collected and used to establish homozygous stocks. As described above, the *roX2* transgene (*UAS-roX2*) was inserted into the X chromosome, because its expression from an autosome causes assembly of the MSL complex at the expression site and ectopic upregulation of the surrounding autosomal genes^30^.

### Transcriptome analysis

For RNA-seq of female and male PGCs at stages 12–16, 1.9–2.3 × 10^5^ female and male PGCs (Table S2) were isolated by fluorescence-activated cell sorting (FACS), as described^21,31^, from 8 to 16 h AEL embryos derived from *vasa-EGFP*/*vasa-EGFP*; *nos-Gal4*/*nos-Gal4* females mated with *UAS*-*RFP*/Y males (Fig. S1a). EGFP and RFP double-positive cells and EGFP-positive cells were isolated as female and male PGCs, respectively. Total RNA was extracted from isolated PGCs using the RNeasy mini Kit (QIAGEN, 74104). Library creation using the TruSeq Standard mRNA Library Prep Kit (Illumina, 20020594) and RNA-seq using the HiSeq 2500 platform (Illumina) were carried out at the University of Minnesota Genomics Center (UMGC), and approximately 40 million reads per sample (125-bp paired-end reads) were obtained.

For RNA-seq of PGCs at stages 15–16, one hundred each of female and male PGCs were isolated from 12 to 16 h AEL embryos derived from *vasa-EGFP*/*vasa-EGFP*; *nos-Gal4*/*nos-Gal4* females mated with *UAS*-*RFP*/Y males (Fig. S1a). For RNA-seq of stages 15–16 *msl* oe male PGCs overexpressing *msl-1*, *msl-2*, *msl-3*, and *roX2*, 100 male PGCs were isolated from 12 to 16 h AEL embryos derived from *UAS-msl-3 UAS-roX2*/*UAS-msl-3 UAS-roX2*; *vasa-EGFP*/*vasa-EGFP*; *nos-Gal4*/*nos-Gal4* females mated with *UAS*-*RFP*/Y; *UAS-msl-1 UAS-msl-2*/*UAS-msl-1 UAS-msl-2* males (Fig. S1b). cDNAs were synthesized using the SMART-Seq v4 Ultra Low Input RNA Kit for Sequencing (Clontech, 634890). Nextera XT library creation and RNA-seq were carried out at UMGC using the HiSeq 2500 platform, and approximately 20 million reads per sample (50-bp paired-end reads) were obtained.

Raw reads obtained from both stage 12–16 and stage 15–16 samples were processed using Trimmomatic-0.36^32^, and then aligned to the transcript model of *Drosophila melanogaster* (Flybase; dmel-all-transcript-r6.23.fasta) using Kallisto-0.44.1 with default settings^33^. Using edgeR^34^, TPM (transcripts per million) in each sample and fold change (log_2_ expression ratios) of each transcript were calculated in female PGCs and *msl* oe male PGCs relative to male PGCs and in female PGCs relative to *msl* oe male PGCs. The raw fastq files obtained from stage 12–16 and stage 15–16 samples were deposited in DDBJ Bio Project database under Accession No. DRA010933 and No. DRA10934, respectively.

### Microarray data analysis

To compare gene expression profiles of somatic tissues between males and females, we used microarray data of thoraxes dissected from adult males and females^11^. These data have been deposited in the Gene Expression Omnibus (GEO) under accession No. GSE30850. Raw values were normalized across samples using quantile normalization, and fold changes in female vs. male thorax were calculated for each gene.

To select zygotically expressed genes, we used microarray data obtained from PGCs at 11 different embryonic stages^21^. These data have been deposited in GEO under Accession No. GSE83460. Raw values were normalized across samples using quantile normalization. Genes expressed at low levels in PGCs at stage 4 (log_2_ expression values < 7) and at high levels in PGCs at stage 16 (log_2_ expression values > 8) were selected as zygotically expressed genes.

### Fixation of embryos

For immunostaining and in situ hybridization, embryos were collected and dechorionated in a sodium hypochlorite solution. The dechorionated embryos were fixed in 1:1 heptane:fixative [4% paraformaldehyde in PBS (130 mM NaCl, 7 mM Na_2_HPO_4_, and 3 mM NaH_2_PO_4_)] for 30 min. Vitelline membranes of the fixed embryos were removed by vigorous shaking in 1:1 methanol:heptane. The embryos were then rinsed with methanol and stored in methanol at − 20 °C until use.

Testes were dissected from adult flies 3–5 days after eclosion and fixed for 15 min. Fixed testes were rinsed with PBSTr (PBS containing 0.1% Triton X-100) and stored in PBSTr at 4 °C until use.

### Immunostaining

Immunofluorescence staining of embryos was performed as described^35,36^. The following experiments were conducted at room temperature unless otherwise stated. *y w* and *nos-Gal4* embryos and embryos derived from *UAS-msl-3 UAS-roX2*/*UAS-msl-3 UAS-roX2*; *nos-Gal4*/*nos-Gal4* females mated with *UAS-msl-1 UAS-msl-2*/*UAS-msl-1 UAS-msl-2* males were fixed as described. Fixed embryos were incubated with 7:3, 5:5, and 3:7 methanol:PBSTr for 5 min each, and then washed with PBSTr three times for 15 min each. The embryos were then incubated with blocking solution (PBS containing 2% BSA, 0.1% Tween 20, and 0.1% Triton X-100) for 1 h. After blocking, the embryos were incubated overnight at 4 °C with rabbit anti-H4K16ac (1:200; Merck, 07–329) and chick anti-Vasa (1:500)^37^. The embryos were washed with PBSTr three times for 15 min each, and then incubated overnight at 4 °C with Alexa Fluor 488–conjugated goat anti-rabbit (1:500; Thermo Fisher Scientific, A11034) and Alexa Fluor 633–conjugated goat anti-chick (1:500; Thermo Fisher Scientific, A21071). The embryos were washed with PBSTr three times for 15 min each, and mounted in VECTASHIELD Mounting Medium (VECTOR, H-1000). The sex of each embryo was determined based on H4K16ac staining in the soma.

Immunofluorescence staining of testes was performed as described^35,36^. The following experiments were conducted at room temperature unless otherwise stated. Testes of progeny from *nos-Gal4*/*nos-Gal4* females mated with *y w* males and *UAS-msl-3 UAS-roX2*/*UAS-msl-3 UAS-roX2*; *nos-Gal4*/*nos-Gal4* females mated with *UAS-msl-1 UAS-msl-2*/*UAS-msl-1 UAS-msl-2* males were fixed as described. Fixed testes were washed within PBSTr three times for 15 min each, and incubated with blocking solution for 1 h. After blocking, the testes were incubated overnight at 4 °C with chick anti-Vasa (1:500)^37^. The testes were washed with PBSTr three times for 15 min each, incubated overnight at 4 °C with Alexa Fluor 488–conjugated goat anti-chick (1:500, Thermo Fisher Scientific, A11039), washed with PBSTr three times for 15 min each, and mounted in VECTASHIELD Mounting Medium.

### Double staining for in situ hybridization and immunostaining

To synthesize RNA probes for in situ hybridization, total RNA was isolated from 4 to 8 h AEL embryos, and cDNA was synthesized using Superscript III. cDNAs corresponding to *msl-1*, *msl-2*, *msl-3*, *mof*, *mle*, *roX1*, and *roX2* were amplified using the following primer pairs: msl-1IHFw/msl-1IHRv, msl-2IHFw/msl-2IHRv, msl-3IHFw/msl-3IHRv, mofIHFw/mofIHRv, mleIHFw/mleIHRv, roX1IHFw/roX1IHRv, and roX2IHFw/roX2IHRv, respectively (Table S1). Amplified cDNAs were cloned into pGEM-T Easy (Promega, A1360). Templates for RNA probes were amplified from these plasmids using the T7 and SP6 primers, and digoxigenin (DIG)-labeled RNA probes were synthesized from the fragments using T7 and SP6 RNA polymerase (Merck, 10881767001 and 10810274001), respectively.

Whole-mount in situ hybridization combined with immunostaining was performed as described^21^. The following experiments were conducted at room temperature unless otherwise stated. Fixed embryos derived from *nos-Gal4*/*nos-Gal4* females mated with *UAS*-*RFP*/Y males were rinsed with ME [50 mM EGTA (pH 8.0) in 90% methanol] and incubated in 7:3, 5:5, and 3:7 ME:fixative for 5 min each. The embryos were re-fixed with fixative for 20 min, and then washed with PBSTw (PBS containing 0.1% Tween 20) three times for 5 min each. The embryos were digested with 50 µg/ml Proteinase K in PBSTw for 3 min, and the digestion was stopped by incubation in fixative for 20 min. The digested embryos were washed with PBSTw three times for 10 min each, and then incubated in pre-hybridization solution [pre-HS; 50% formamide, 5 × SSC (750 mM NaCl and 75 mM sodium-citrate), 100 µg/ml heparin, 100 µg/ml yeast tRNA, 10 mM DTT, and 0.1% Tween 20] for 60 min at 60 °C. They were then hybridized with 2 ng/µl RNA probe in hybridization solution (pre-HS containing 10% dextran sulfate) overnight at 60 °C. After hybridization, the embryos were washed with a washing solution (50% formamide, 5 × SSC, and 0.1% Tween 20) six times for 30 min each at 60 °C. The embryos were rinsed with TNT [100 mM Tris–HCl (pH 7.5), 150 mM NaCl, and 0.05% Tween 20] three times and incubated with TNB [0.5% blocking reagent (PerkinElmer, NEL700001KT), 100 mM Tris–HCl (pH 7.5), and 150 mM NaCl)] for 30 min. After blocking, the embryos were incubated with TNB containing a horseradish peroxidase–conjugated anti-DIG antibody (1:250; Merck, 11,633,716,001) for 30 min, and then washed with TNT three times for 15 min each. The embryos were incubated in Amplification diluent containing biotin tyramide solution (1:50; PerkinElmer, NEL700001KT) for 20 min, and then washed with TNT three times for 15 min each. The embryos were incubated overnight at 4 °C in TNB containing chick anti-Vasa (1:500)^37^ and rabbit anti-RFP (1:1000; Thermo Fisher Scientific, R10367) and washed with TNT three times for 15 min each. For detection of RNA probes and antibodies, embryos were incubated overnight at 4 °C in TNB containing fluorescein-conjugated streptavidin (1:1000; PerkinElmer, S869), Alexa Fluor 633–conjugated goat anti-chick (1:500, Thermo Fisher Scientific, A21103), and Alexa Fluor 546–conjugated goat anti-rabbit (1:500, Thermo Fisher Scientific, A11035). The embryos were washed with TNT three times for 15 min each, and then mounted in VECTASHIELD Mounting Medium. The sex of each embryo was determined based on RFP staining, except in the case of stage 5 embryos.

## Results and discussion

### Dosage compensation is indiscernible in the PGCs

**Figure 1:**
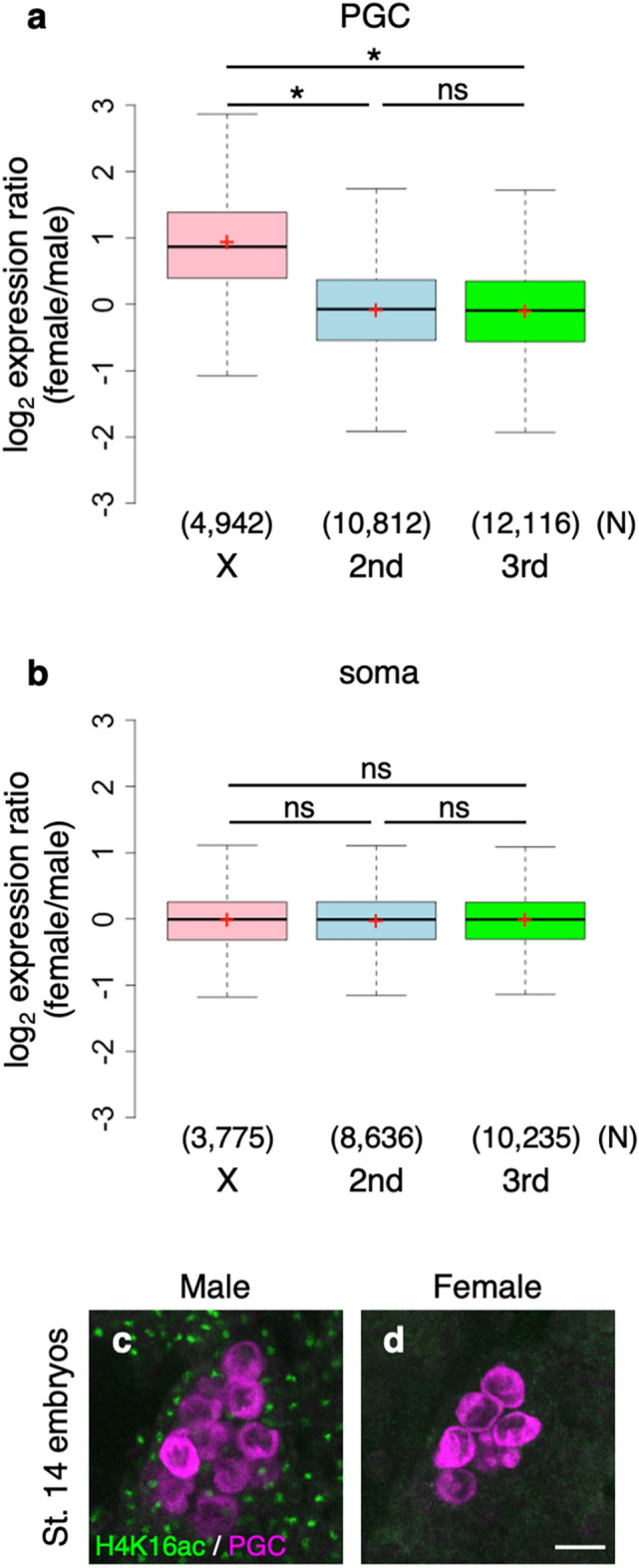
Dosage compensation is absent in embryonic PGCs. (**a**) Log_2_ expression ratio of transcripts from genes on the X (pink), second (light blue), and third chromosomes (lime green) between female and male PGCs (female/male) at embryonic stages 12–16. Each box plot represents the median value and first and third quartile values. Error bars represent minimum and maximum values. Red plus signs represent mean values. Outliers are not depicted. The mean values for the transcripts from genes on the X, second, and third chromosomes were 0.94, − 0.08, and − 0.10, respectively. Significance was calculated by two-sided Mann–Whitney U test (*: *P* < 0.05, ns: not significant). Transcripts for which TPM were zero in all samples were excluded from this analysis. N: number of the transcripts examined. (**b**) Log_2_ expression ratio of transcripts from genes on the X (pink), second (light blue), and third chromosomes (lime green) between female and male thoraxes (female/male) dissected from adults. This result was obtained from microarray data deposited by Meiklejohn et al. (GEO accession No. GSE30850)^11^. Each box plot represents values as in (**a**). The mean values for the transcripts from genes on the X, second, and third chromosomes were − 0.01, − 0.02, and − 0.01, respectively. Significance was calculated by two-sided Mann–Whitney U test (ns: not significant). N: number of the probes examined. (**c** and **d**) H4K16ac expression in male (**c**) and female (**d**) PGCs at embryonic stage 14 (St. 14). *y w* embryos were stained for H4K16ac (green) and Vasa [a marker of germline cells (magenta)]. Embryos were sexed based on H4K16ac staining in the soma. Scale bar: 10 µm.

To investigate whether dosage compensation occurs in male PGCs, we separately isolated female and male PGCs and compared the expression of X-linked genes between the two sexes. For this purpose, we used females carrying *nos-Gal4* and *vasa-EGFP* mated with males carrying *UAS-RFP* on the X chromosome^21^ (Fig. S1a). In embryos derived from these mothers, female and male PGCs were double-positive for EGFP and RFP and single-positive for EGFP, respectively (Fig. S1a), as *nos-Gal4*–driven *UAS-RFP* is activated only in female PGCs from stage 9 onward, whereas *vasa-EGFP* is expressed throughout germline development in both sexes^25,26,37^. RNA-seq data were obtained from female and male PGCs at stages 12–16. Our data revealed that female-biased expression was significantly more common in transcripts from X-linked genes than in those from autosomal genes, although sex-biased expression was also observed in autosomes (Fig. S2a–f). Expression of the transcripts from X-linked genes was twofold higher on average in female PGCs than in male PGCs, whereas the female/male expression ratio of transcripts from autosomal genes was ~ 1 (Fig. 1a). Similar female-biased expression was observed when zygotically expressed genes were selected from X-linked genes (Fig. S3), indicating female-biased zygotic expression from X-linked genes. In addition, we found that expression of X-linked housekeeping genes for glycolysis and ATP synthesis was female-biased in PGCs (Fig. S4). By contrast, in the soma, female-biased expression of X-linked genes was not observed (Fig. 1b). In addition, we found that H4K16ac was undetectable in the PGCs, which were surrounded by H4K16ac-positive somatic cells in males (Fig. 1c,d). These observations led us to conclude that expression of genes on the X chromosome is subjected to dosage compensation in the soma, but not in PGCs. However, we cannot rule out the possibility that expression of a fraction of X-linked genes in male PGCs is compensated by mechanisms independent of H4K16ac.

Zygotic expression of *ovo* and *ovarian tumor* (*otu*) genes on the X chromosome is female-biased^38^, and both genes are required for female germline development^20,39,40^. We found that these genes exhibited female-biased expression (Fig. S2d). Furthermore, transcripts from marker genes for male PGCs^38^, such as *disc proliferation abnormal* (*dpa*), *CG9253*, *no child left behind* (*nclb*), *Ran GTPase activating protei*n (*RanGap*), *Rs1*, *CG6693*, *Kinesin-like protein at 61F* (*Klp61F*), and *Minichromosome maintenance 5* (*Mcm5*), exhibited male-biased expression in PGCs (Fig. S2e and f).

### Expression of genes encoding the components of MSL complex in male PGCs during embryogenesis

**Figure 2:**
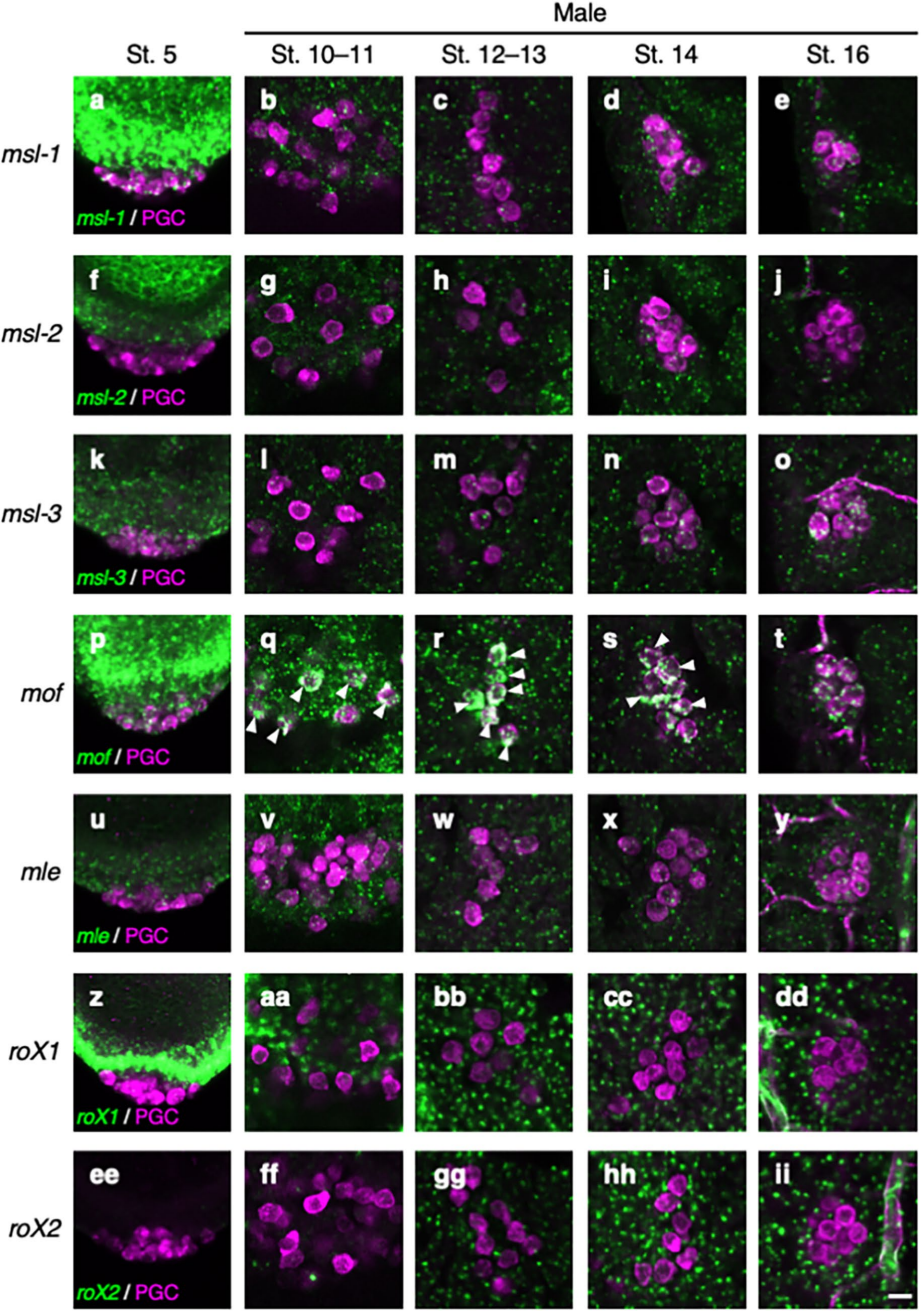
Expression of the transcripts encoding the components of MSL complex in male PGCs during embryogenesis. RNA expression from *msl-1* (**a**–**e**), *msl-2* (**f**–**j**), *msl-3* (**k**–**o**), *mof* (**p**–**t**), *mle* (**u**–**y**), *roX1* (**z**–**dd**), and *roX2* (**ee**–**ii**) in PGCs at embryonic stage 5 (St. 5; a, f, k, p, u, z, and ee) and in male PGCs at embryonic stages 10–11 (St. 10–11; b, g, l, q, v, aa, and ff), stages 12–13 (St. 12–13; c, h, m, r, w, bb, and gg), stage 14 (St. 14; d, i, n, s, x, cc, and hh), and stage 16 (St. 16; e, j, o, t, y, dd, and ii). Embryos derived from *nos-Gal4*/*nos-Gal4* females mated with *UAS-RFP*/Y males were in situ hybridized with a probe for each gene (green) and immunostained for Vasa (magenta) and RFP. Because *nos-Gal4* activates *UAS-RFP* only in female PGCs from stage 9 onward, the sexes of PGCs could be determined by RFP signal at stages 10–16. Probes were designed to detect all RNA variants identified in each gene region. Scale bar: 10 µm. White arrowheads show PGCs with high-level signals (green).

Given that the MSL complex is required for dosage compensation of the X chromosome through H4K16ac in the soma^4,5^, we next asked whether genes encoding the components of the MSL complex are expressed in male PGCs during embryogenesis. For this purpose, we performed in situ hybridization of embryos produced from females carrying *nos-Gal4* mated with males carrying *UAS-RFP* on the X chromosome. In the PGCs of stage 5 embryos, *msl1* and *mof* mRNAs were detectable (Fig. 2a and p). Expression of these mRNAs is considered to be maternal in origin, as PGCs are transcriptionally repressed at this stage^26,41,42^, and their zygotic transcription in PGCs is repressed until late gastrulation. Furthermore, we detected the *msl-2*, *msl-3*, and *mle* mRNAs and two *roX* RNAs at very low levels (Fig. 2f, k, u, z, and ee). In PGCs at the middle and late embryonic stages (stages 10–16), only *mof* mRNA was observed at a high level (Fig. 2q–t), and the *msl-1*, *msl-2*, *msl-3*, and *mle* mRNAs and two *roX* RNAs were all detected at low levels, if at all (Fig. 2b–e, g–j, l–o, v–y, aa–dd, and ff–ii). We observed no significant difference in the expression of these RNAs between male and female PGCs (Fig. 2 and Fig. S5). These observations suggest that the PGCs are depleted of some essential components of the MSL complex, which in turn eliminates the ability to induce dosage compensation in male PGCs.

### Overexpression of msl-1, msl-2, msl-3, and roX2 induces H4K16ac and hyperactivation of X-linked genes in male PGCs

**Figure 3:**
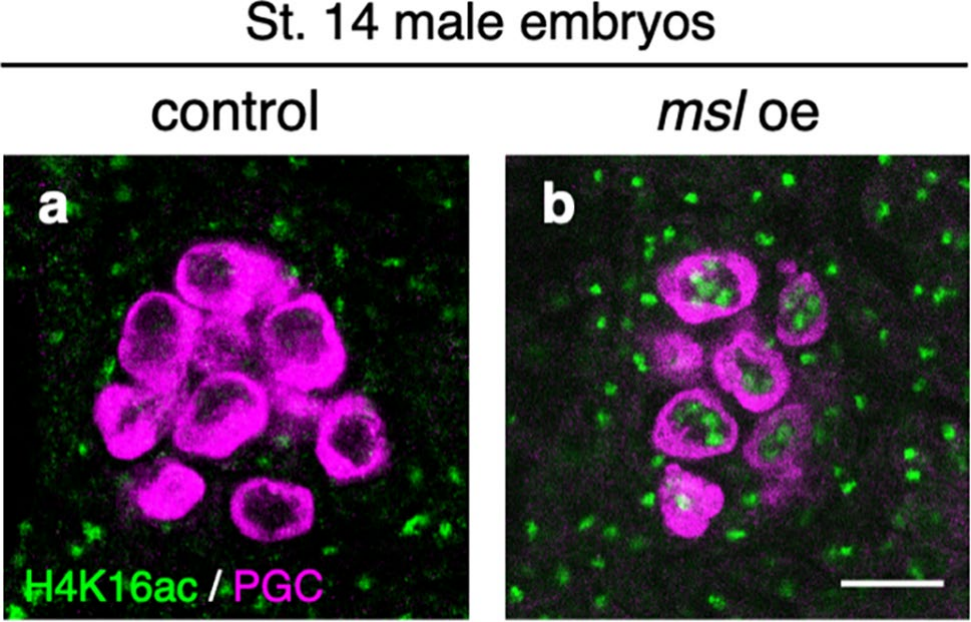
Overexpression of *msl-1*, *msl-2, msl-3*, and *roX2* induces H4K16ac in male PGCs. (**a** and **b**) H4K16ac expression in male PGCs of *nos-Gal4* strain (**a**; control) and male PGCs overexpressing *msl-1*, *msl-2, msl-3*, and *roX2* (**b**; *msl* oe) at embryonic stage 14. Embryos were stained for H4K16ac (green) and Vasa (magenta). Embryos were sexed based on H4K16ac staining in the soma. Scale bar: 10 µm.

To determine whether depletion of MSL components causes deficiency of dosage compensation in male PGCs, we overexpressed genes encoding MSL components in male PGCs. MLE protein is maternally supplied and expressed in male PGCs during embryogenesis, whereas MSL-1 and MSL-2 proteins are not^43^. Hence, we forced expression of *msl-1*, *msl-2*, *msl-3*, and *roX2* in PGCs. Only *roX2* was overexpressed because the *roX* RNAs are functionally redundant^44^. When *msl-1*, *msl-2*, *msl-3*, and *roX2* were overexpressed under the control of *nos-Gal4* (*msl* oe), H4K16ac became detectable in the nuclei of male PGCs (Fig. 3a,b).

**Figure 4:**
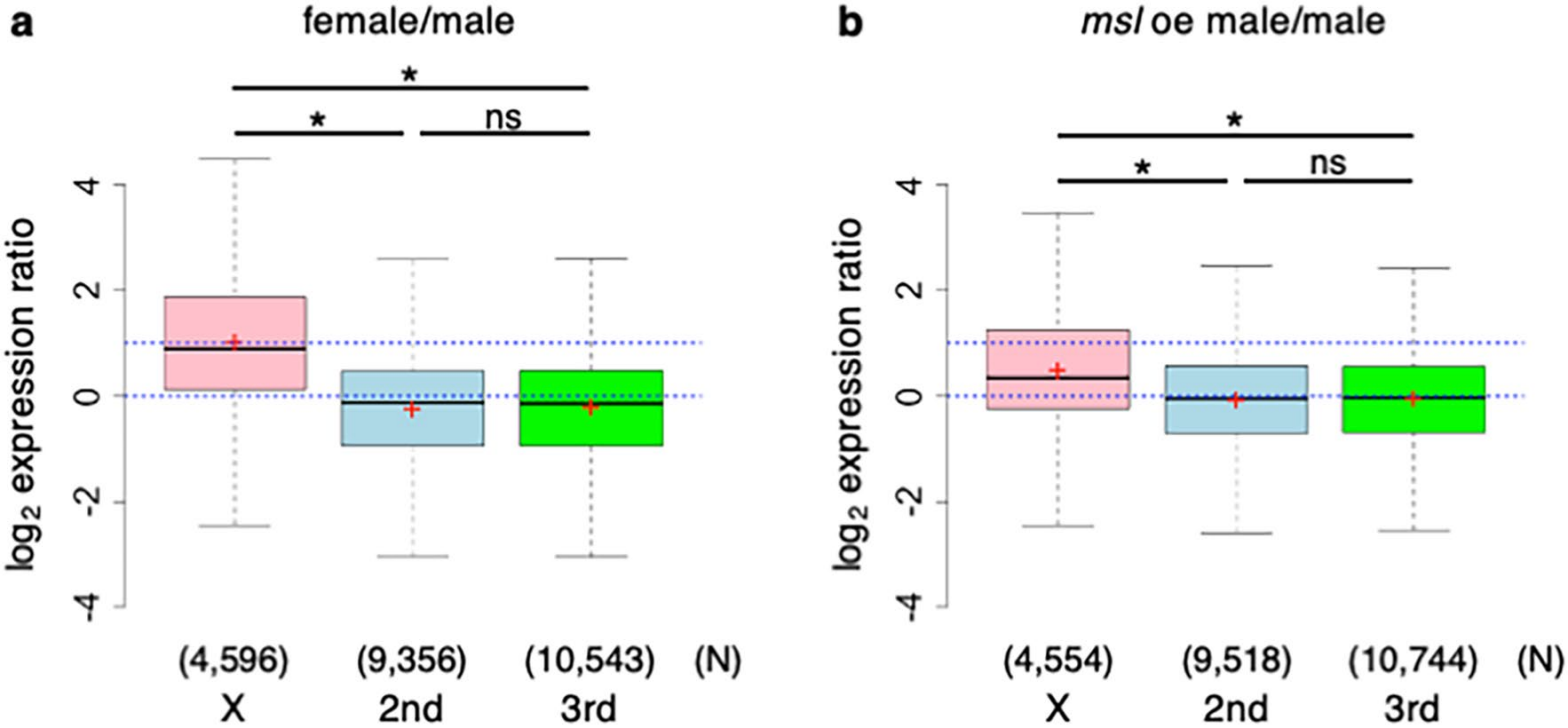
Overexpression of *msl-1*, *msl-2, msl-3*, and *roX2* induces hyperactivation of X-linked genes in male PGCs. (**a**) Log_2_ expression ratio of transcripts from genes on the X (pink), second (light blue), and third chromosomes (lime green) between female and male PGCs (female/male) at embryonic stages 15–16. Each box plot represents values as in Fig. 1a. Mean values for the X, second, and third chromosomes were 1.03, − 0.23, and − 0.21, respectively. Blue dotted lines indicate log_2_ expression ratios of 0 and 1. Significance was calculated by two-sided Mann–Whitney U test (*, *P* < 0.05; ns, not significant). Transcripts for which TPM was zero in all samples were excluded from this analysis. N: number of the transcripts examined. (**b**) Log_2_ expression ratio of transcripts from genes on the X (pink), second (light blue), and third chromosomes (lime green) between *msl* oe male PGCs and male PGCs (*msl* oe male/male) at embryonic stages 15–16. Each box plot represents values as in Fig. 1a. Mean values for the X, second, and third chromosomes were 0.51, − 0.06, and − 0.04, respectively. Blue dotted lines indicate log_2_ expression ratios of 0 and 1. Significance was calculated by two-sided Mann–Whitney U test (*, *P* < 0.05; ns, not significant). Transcripts for which TPM was zero in all samples were excluded from this analysis. N: number of transcripts examined. Expression ratio of the autosomal genes in *msl* oe male/male was higher than in female/male in (**a**) (*P* values, calculated by two-sided Mann–Whitney U test for the second and third chromosomes, were < 0.05), but the effect sizes calculated by Cliff’s Delta for the second and third chromosomes were statistically negligible (0.07 and 0.08, respectively)^51^. Expression ratio of X-linked genes in *msl* oe male/male was significantly lower than in female/male in (**a**) (*P* values, calculated by two-sided Mann–Whitney U test, were < 0.05), and the effect size was non-negligible (0.21).

We next asked whether overexpression of MSL components would result in hyperactivation of X-linked genes in male PGCs. We performed RNA-seq analysis of female and male PGCs and male PGCs overexpressing *msl-1*, *msl-2*, *msl-3*, and *roX2* at stages 15–16. We found that X-linked genes exhibited female-biased expression relative to autosomal genes at stages 15–16 (Fig. S6a–c and Fig. S7a–c), and that their expression was twofold higher in female PGCs than in male PGCs (Fig. 4a). These data, obtained from PGCs at stages 15–16, were similar to those obtained from PGCs at stages 12–16 (Fig. 1a). When *msl-1*, *msl-2*, *msl-3*, and *roX2* were overexpressed in male PGCs (*msl* oe male), X-linked genes were significantly hyperactivated relative to male PGCs (Fig. 4a,b, Fig. S6d–f, Fig. S7d–f, and Fig. S8). Autosomal genes were also activated, but the degree of upregulation was statistically negligible (Fig. 4a,b). These results indicate that overexpression of *msl-1*, *msl-2*, *msl-3*, and *roX2* in male PGCs can hyperactivate X-linked genes. Thus, failure of MSL complex formation resulting from low or no expression of *msl-1*, *msl-2*, *msl-3*, and *roX* RNAs causes the lack of dosage compensation in male PGCs. Future studies focusing on the regulatory mechanisms of *msl-1*, *msl-2*, *msl-3*, and *roX* expression will represent an important step toward elucidating how dosage compensation is repressed in male PGCs.

Although hyperactivation of X-linked genes was evident in male PGCs overexpressing *msl-1*, *msl-2*, *msl-3*, and *roX2* (Fig. 4a,b, Fig. S6d–f, Fig. S7d–f, and Fig. S8), the upregulation ratio was only ~ 1.5-fold (Fig. 4b). Thus, overexpression of *msl-1*, *msl-2*, *msl-3*, and *roX2* in male PGCs is insufficient to induce twofold upregulation of X-linked genes, as observed in male soma (Fig. 1b). One possible explanation for this is that dosage compensation requires factors other than the MSL complex in male PGCs. The functions of *JIL-1 kinase* and *Topoisomerase 2* are also needed for dosage compensation in male soma^45–47^. The possibility that overexpression of these molecules, along with *msl-1*, *msl-2*, *msl-3*, and *roX2*, can induce twofold upregulation of X-linked genes in male PGCs remains to be tested.

### Biological significance of the absence of dosage compensation in male PGCs

In *Drosophila*, the sexual identity of the germline is regulated by both cell-autonomous cues, which are produced depending on X-chromosome constitution, and the sex of the surrounding soma^23,48–50^. In the absence of dosage compensation, X-linked genes are expressed at twofold higher levels in female (XX) PGCs than in male (XY) PGCs at stages 12–16 (Fig. 1a). This biased expression of X-linked genes may be one of the determinants of femaleness. We found that overexpression of *msl-1*, *msl-2*, *msl-3*, and *roX2* can hyperactivate X-linked genes in XY PGCs (Fig. 4a,b, Fig. S6d–f, Fig. S7d–f, and Fig. S8), suggesting that XY PGCs acquire femaleness, but only partially. Indeed, overexpression of *msl-1*, *msl-2*, *msl-3*, and *roX2* in XY PGCs caused upregulation of *ovo* (Fig. S7d) and downregulation of genes that exhibited male-biased expression in PGCs (Fig. S7e and f). Thus, it is possible that female sexual identity of PGCs can be brought about by introducing dosage compensation. However, PGCs overexpressing *msl-1*, *msl-2*, *msl-3*, and *roX2* executed normal spermatogenesis and became functional sperm (Fig. S9). This is presumably because masculinizing signals from the surrounding male soma^38^ override feminization of PGCs. Transplantation of XY PGCs overexpressing *msl-1*, *msl-2*, *msl-3*, and *roX2* into female soma may clarify whether twofold upregulation of X-lined genes induces femaleness in XY PGCs.

## Supplementary Information

Supplementary Information

## Data Availability

All data and materials produced by this study are available from the corresponding authors upon reasonable request.
