# Supplementary material for: Absence of X-chromosome dosage compensation in the primordial germ cells of Drosophila embryos
Source: Sci Rep. 2021 Mar 1;11:4890. doi: 10.1038/s41598-021-84402-7 (PMC7921590; doi:10.1038/s41598-021-84402-7)
Supplement: Supplementary file 1 — Supplementary Information [file 41598_2021_84402_MOESM1_ESM.pdf]

Absence of X-chromosome dosage compensation in the primordial germ cells of *Drosophila* embryos

Ryoma Ota[1,2,3*], Makoto Hayashi[3,4], Shumpei Morita[3,4,5], Hiroki Miura[4], and Satoru Kobayashi[3,4*]

[1]Department of Biosciences, Faculty of Science and Engineering, Teikyo University, Utsunomiya, Tochigi, 320-8551, Japan
[2]Division of Integrated Science and Engineering, Graduate School of Science and Engineering, Teikyo University, Utsunomiya, Tochigi, 320-8551, Japan
[3]Life Science Center for Survival Dynamics, Tsukuba Advanced Research Alliance (TARA), University of Tsukuba, Tsukuba, Ibaraki, 305-8577, Japan
[4]Graduate School of Life and Environmental Sciences, University of Tsukuba, Tsukuba, Ibaraki, 305-8577, Japan
[5]Present Address: Molecular Biology, Cell Biology and Biochemistry, Brown University, Providence, RI 02906, USA

[*]Corresponding author:
Ryoma Ota; ota@nasu.bio.teikyo-u.ac.jp, Tel: +81-028-627-7145
Satoru Kobayashi; skob@tara.tsukuba.ac.jp, Tel: +81- 029-853-5881

Supplementary Figure S1

a

Genotypes of parents

♀♀ + / + ; *vasa-EGFP* / *vasa-EGFP* ; *nos-Gal4* / *nos-Gal4* × ♂♂ *UAS-RFP* / Y ; + / + ; + / +

Genotype of female progeny

UAS-RFP / + ; *vasa-EGFP* / + ; *nos-Gal4* / +

Genotype of male progeny

Y / + ; *vasa-EGFP* / + ; *nos-Gal4* / +

b

Genotypes of parents

♀♀ *UAS-msl-3* *UAS-roX2* / *UAS-msl-3* *UAS-roX2* ; *vasa-EGFP* / *vasa-EGFP* ; *nos-Gal4* / *nos-Gal4* × ♂♂ *UAS-RFP* / Y ; *UAS-msl-1* *UAS-msl-2* / *UAS-msl-1* *UAS-msl-2* ; + / +

Genotype of female progeny

UAS-RFP / *UAS-msl-3* *UAS-roX2* ; *vasa-EGFP* / *UAS-msl-1* *UAS-msl-2* ; *nos-Gal4* / +

Genotype of male progeny (*msl* oe male)

Y / *UAS-msl-3* *UAS-roX2* ; *vasa-EGFP* / *UAS-msl-1* *UAS-msl-2* ; *nos-Gal4* / +

Fig. S1: Genotypes of flies used for the isolation of female and male PGCs and male PGCs overexpressing *msl-1*, *msl-2*, *msl-3*, and *roX2* (*msl* oe male).

(a) Genotypes of parents (upper) and female (middle) and male (lower) progeny used for the isolation of female and male PGCs. Plus signs and Y represent chromosomes carrying no transgenes and the Y chromosome, respectively. To isolate female and male PGCs, females homozygous for *vasa-EGFP* (on the second chromosome) and *nos-Gal4* (on the third chromosome) were mated with males hemizygous for *UAS-RFP* (on the X chromosome). In progeny derived from these parents, females were heterozygous for *UAS-RFP*, *vasa-EGFP*, and *nos-Gal4* (middle), whereas males were heterozygous for *vasa-EGFP and nos-Gal4* (lower). Because *UAS-RFP* was activated by *nos-GAL4* in only female PGCs, and *vasa-EGFP* is expressed in both sexes, EGFP and RFP double-positive cells and EGFP single-positive cells were isolated as female and male PGCs, respectively. **(b)** Genotypes of parents (upper) and female (middle) and male (lower) progeny used for the isolation of *msl* oe male PGCs. To isolate *msl* oe male PGCs, females homozygous for *UAS-msl-3* and *UAS-roX2* (on the X chromosome), *vasa-EGFP* (on the second chromosome), and *nos-Gal4* (on the third chromosome) were mated with males hemizygous for *UAS-RFP* (on the X chromosome) and homozygous for *UAS-msl-1* and *UAS-msl-2* (on the second chromosome). In progeny derived from these parents, males were hemizygous for *UAS-msl-3* and *UAS-roX2* and heterozygous for *UAS-msl-1*, *UAS-msl-2*, *vasa-EGFP*, and *nos-Gal4* (lower). In their male progeny, *UAS-msl-1*, *UAS-msl-2*, *UAS-msl-3*, and *UAS-roX2* were activated by *nos-Gal4* in PGCs and *vasa-EGFP* was expressed in PGCs. Thus, EGFP single-positive cells were isolated as *msl* oe male PGCs.

Supplementary Figure S2

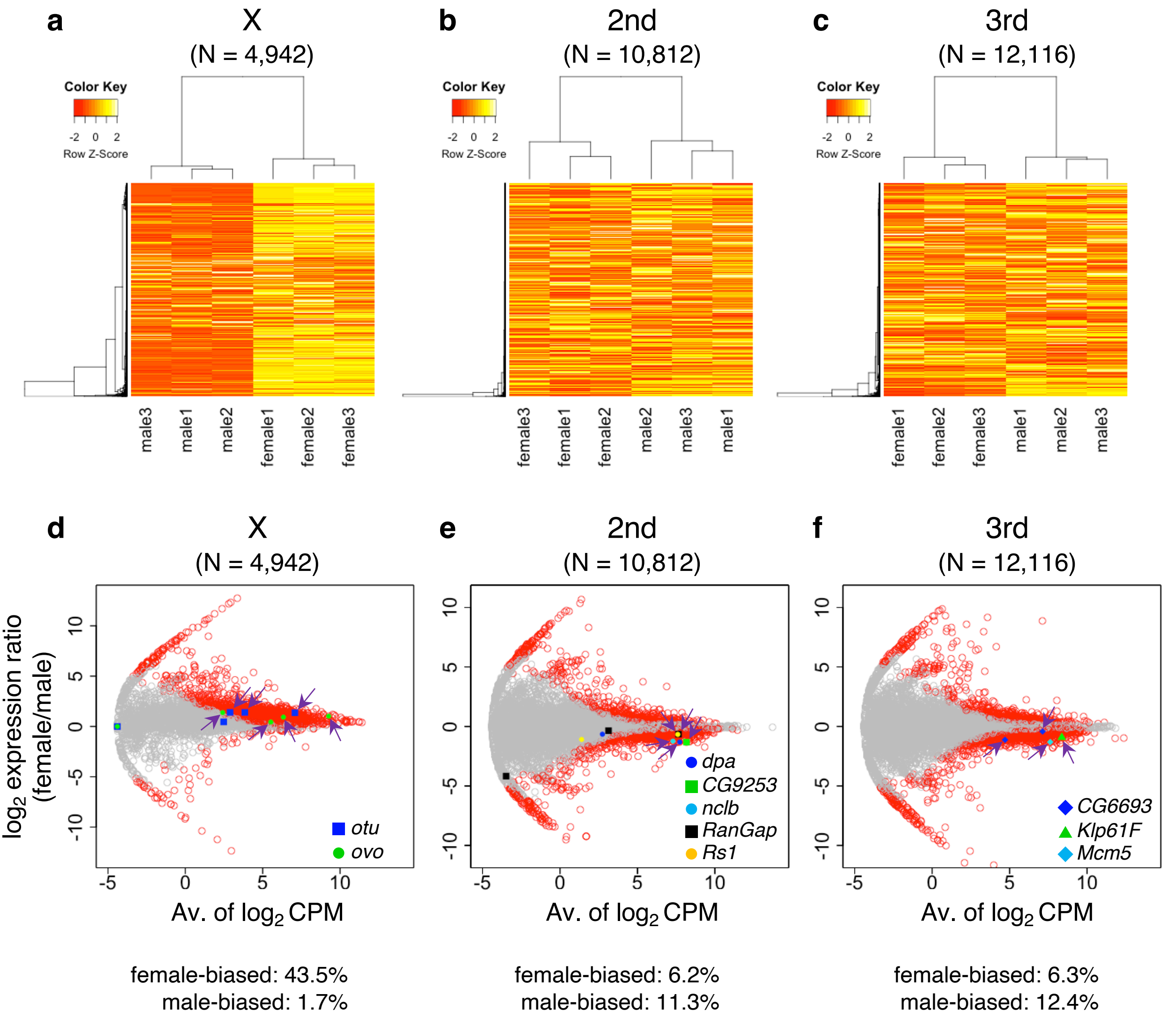

Fig. S2: Heatmaps and MA-plots of transcripts from genes on the X, second, and third chromosomes in female and male PGCs at embryonic stages 12–16.
(a–c) Cluster heatmaps of the transcripts from genes on the X (a), second (b), and third (c) chromosomes in female and male PGCs at embryonic stages 12–16. Estimated counts obtained from raw reads data of each female and male PGCs using Kallisto were subjected to TMM normalization using edgeR. On the basis of the normalized counts, row z-scores (relative expression values) were calculated, and cluster analysis was performed using the gplots package in R. Transcripts for which counts were zero in all samples were excluded from this analysis. N: number of transcripts examined. **(d–f)** MA-plots of transcripts from genes on the X (d), second (e), and third (f) chromosomes. Log_2 expression ratio (female/male) and average of log_2 CPM (count per million) calculated by edgeR were used as the y-axis and x-axis, respectively. Red open circles represent differentially expressed transcripts [False discovery rate (FDR) < 0.05]. Blue squares and green filled circles in (d) represent *otu* and *ovo* transcripts, respectively. Blue filled circles, green square, light blue filled circle, black squares, and yellow filled circle in (e) represent *dpa*, *CG9253*, *nclb*, *RanGap*, and *Rs1* transcripts, respectively. Blue diamonds, green triangle, and light blue diamond in (f) represent *CG6693*, *Klp61F*, and *Mcm5* transcripts, respectively. Purple arrows show differentially expressed transcripts from *ovo*, *otu*, *dpa*, *CG9253*, *nclb*, *RanGap*, *Rs1*, *CG6693*, *Klp61F*, and *Mcm5* (FDR < 0.05). Transcripts for which TPM was zero in all samples were excluded from this analysis. N: the number of transcripts examined. Percentages of transcripts exhibiting female- and male-biased expression (FDR < 0.05) from the X (d), second (e), and third (f) chromosomes are also shown. Female-biased expression was more common transcripts from X-linked genes than in those from autosomal genes (*P* values, calculated by two-sided Fisher's exact test vs. the second and third chromosomes, were < 0.05).

Supplementary Figure S3

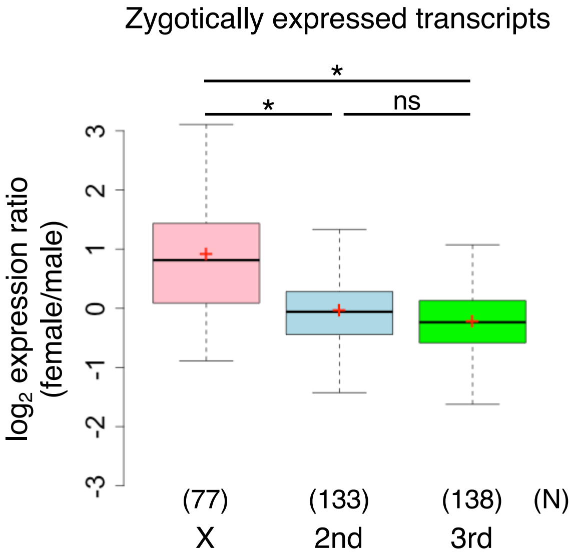

Fig. S3: Log_2 expression ratios of zygotically expressed transcripts from genes on the X, second, and third chromosomes between female and male PGCs.

Log_2 expression ratio of zygotically expressed transcripts from genes on the X (pink), second (light blue), and third chromosomes (lime green) between female and male PGCs (female/male) at embryonic stages 12–16. Based on the microarray data of PGCs, genes expressed at low levels at stage 4 (log_2 expression values < 7) and at high levels at stage 16 (log_2 expression values > 8) were selected as zygotically expressed genes. Each box plot represents values as in Fig. 1a. The mean values for the X, second, and third chromosomes were 0.93, –0.03, and –0.20, respectively. Significance was calculated by two-sided Mann–Whitney U test (*, $P < 0.05$; ns, not significant). Transcripts for which TPM was zero in all samples were excluded from this analysis. N: number of transcripts examined.

Supplementary Figure S4

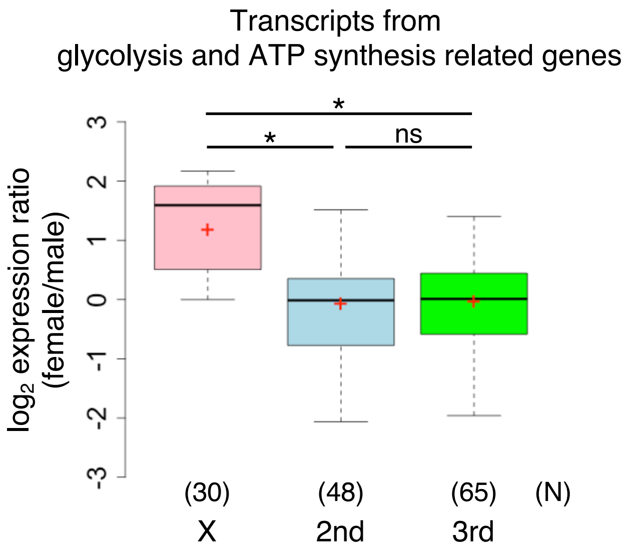

Fig. S4: Log$_2$ expression ratios of transcripts from housekeeping genes on the X, second, and third chromosomes between female and male PGCs.

Log$_2$ expression ratio of transcripts from housekeeping genes on the X (pink), second (light blue), and third chromosomes (lime green) between female and male PGCs (female/male) at embryonic stages 12–16. Genes associated with Gene Ontology terms related to glycolysis and ATP synthesis were selected. Each box plot represents values as in Fig. 1a. The mean values for the X, second, and third chromosomes were 0.94, –0.11, and –0.10, respectively. Significance was calculated by two-sided Mann–Whitney U test (*, $P < 0.05$; ns, not significant). Transcripts for which TPM was zero in all samples were excluded from this analysis. N: number of transcripts examined.

Supplementary Figure S5

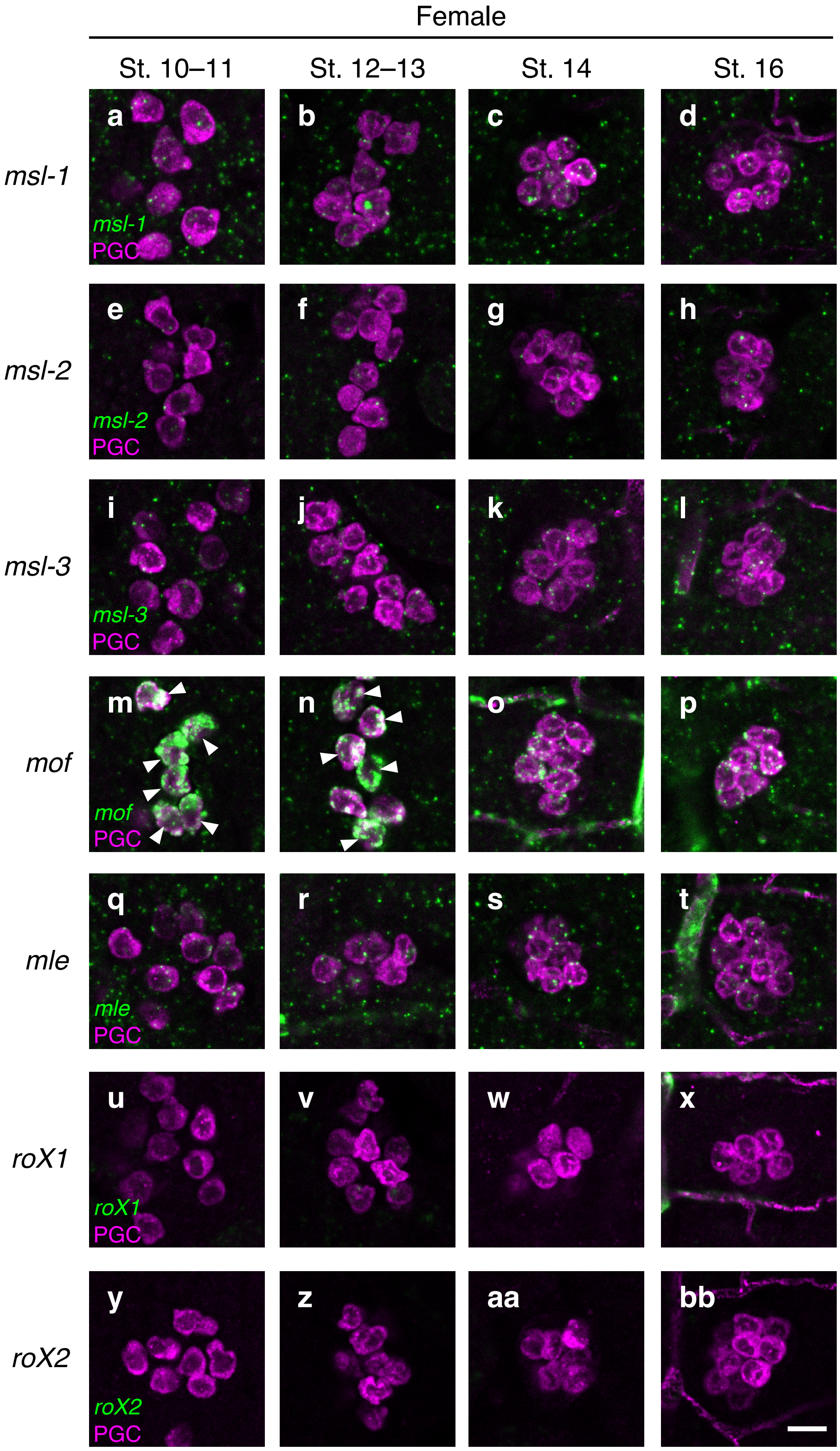

Fig. S5: Expression of transcripts encoding components of the MSL complex in female PGCs during embryogenesis.

RNA expression from *msl-1* (a–d), *msl-2* (e–h), *msl-3* (i–l), *mof* (m–p), *mle* (q–t), *roX1* (u–x), and *roX2* (y–bb) in female PGCs at embryonic stages 10–11 (St. 10–11: a, e, i, m, q, u, and y), stages 12–13 (St. 12–13: b, f, j, n, r, v, and z), stage 14 (St. 14: c, g, k, o, s, w, and aa), and stage 16 (St. 16: d, h, l, p, t, x, and bb). Embryos derived from *nos-Gal4*/*nos-Gal4* females mated with *UAS-RFP*/Y males were *in situ* hybridized with a probe for each gene (green) and immunostained for Vasa (magenta) and RFP. Because *nos-Gal4* activates *UAS-RFP* only in female PGCs from stage 9 onward, the sexes of PGCs can be determined based on the RFP signal at stages 10–16. Probes were designed to detect all RNA variants identified in each gene region. Scale bar: 10 μm. White arrowheads show PGCs with high-level signals (green).

Supplementary Figure S6

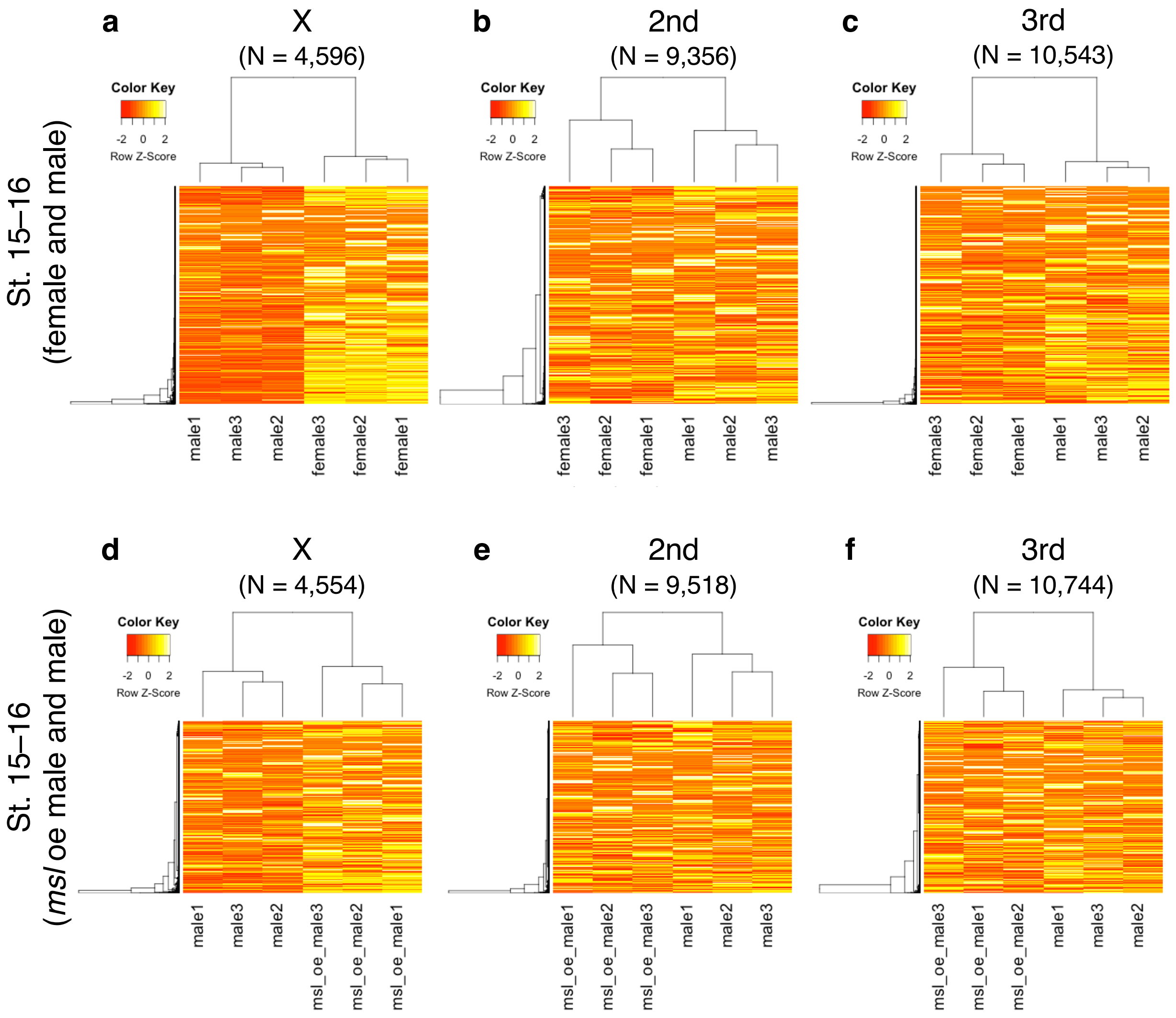

Fig. S6: Heatmaps of transcripts from genes on the X, second, and third chromosomes in PGCs at embryonic stages 15–16.

Cluster heatmaps of the transcripts from genes on the X (a and d), second (b and e), and third (c and f) chromosomes in female and male PGCs (a–c) and in male PGCs overexpressing *msl-1*, *msl-2, msl-3*, and *roX2* (*msl* oe male) and male PGCs (d–f) at embryonic stages 15–16. Estimated counts obtained from raw reads data of female and male PGCs and *msl* oe male PGCs using Kallisto were subjected to TMM normalization using edgeR. On the basis of the normalized counts, row z-scores (relative expression values) were calculated, and cluster analysis was performed using the gplots package in R. Transcripts for which counts were zero in all samples were excluded from each analysis. N: number of transcripts examined.

Supplementary Figure S7

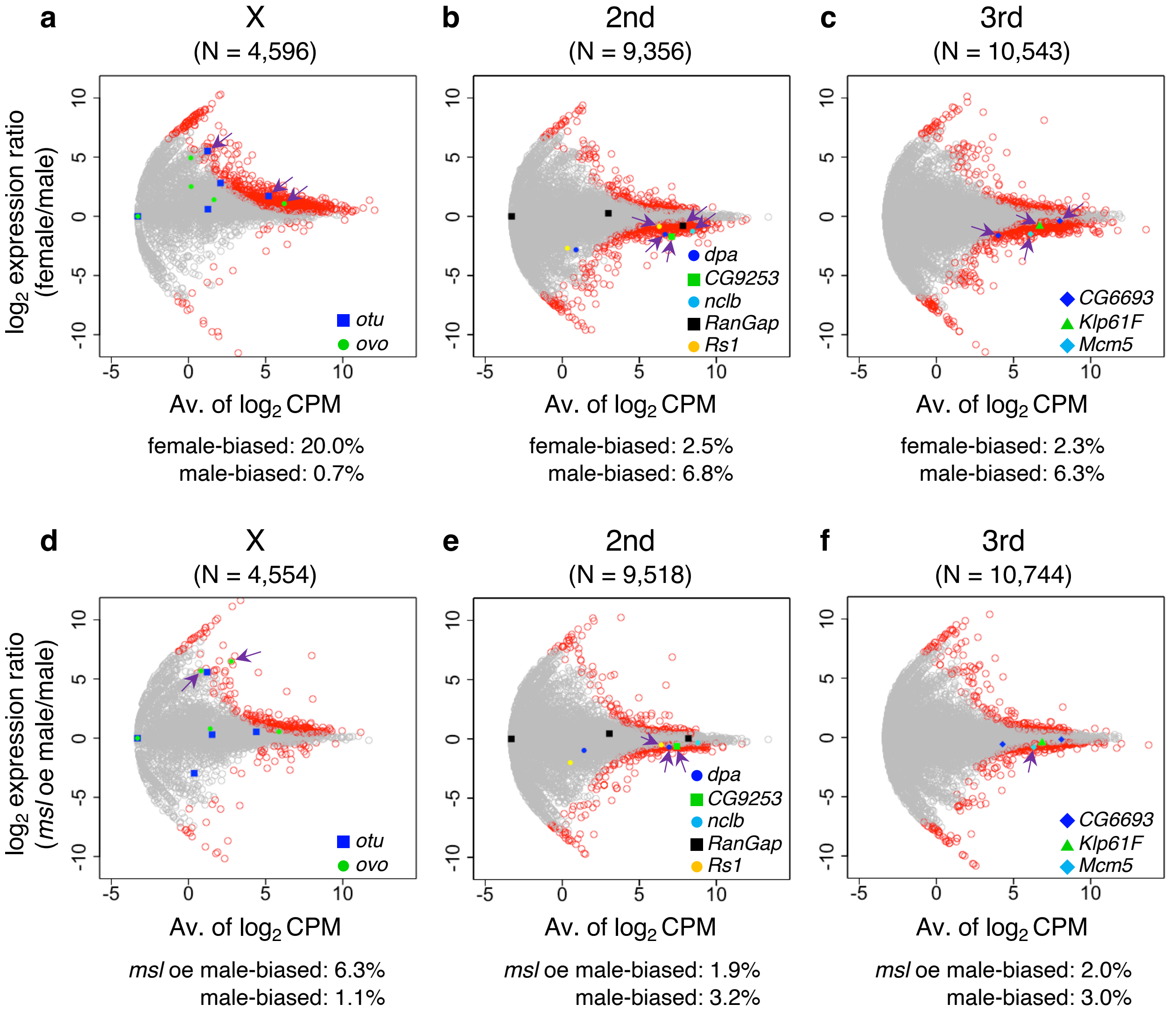

Fig. S7: MA-plots of transcripts from genes on the X, second, and third chromosomes in PGCs at embryonic stages 15–16.
MA-plots of transcripts from genes on the X (a and d), second (b and e), and third (c and f) chromosomes in female and male PGCs (a–c) and in male PGCs overexpressing *msl-1*, *msl-2, msl-3*, and *roX2* (*msl* oe male) and male PGCs (d–f) at embryonic stages 15–16. Log$_2$ expression ratio (female/male in a–c and *msl* oe male/male in d–f) and average log$_2$ CPM calculated by edgeR were used as the y-axis and x-axis, respectively. Red open circles represent differentially expressed transcripts (FDR < 0.05). Blue squares and green filled circles represent *otu* and *ovo* transcripts, respectively, in (a) and (d). Blue filled circles, green square, light blue filled circle, black squares, and yellow filled circle represent *dpa*, *CG9253*, *nclb*, *RanGap*, and *Rs1* transcripts, respectively, in (b) and (e). Blue diamonds, green triangle, and light blue diamond represent *CG6693*, *Klp61F*, and *Mcm5* transcripts, respectively, in (c) and (f). Purple arrows show differentially expressed transcripts from *ovo*, *otu*, *dpa*, *CG9253*, *nclb*, *RanGap*, *Rs1*, *CG6693*, *Klp61F*, and *Mcm5* (FDR < 0.05). Transcripts for which TPM are zero in all samples were excluded from each analysis. N: number of transcripts examined. Percentages of transcripts exhibiting female- and male-biased expression (FDR < 0.05) from the X (a and d), second (b and e), and third (c and f) chromosomes are also shown. Female-based expression (a–c) and *msl* oe male–biased expression (d–f) were more common in transcripts from X-linked genes than from autosomal genes (*P* values, calculated by two-sided Fisher's exact test vs. the second and third chromosomes, were < 0.05).

Supplementary Figure S8

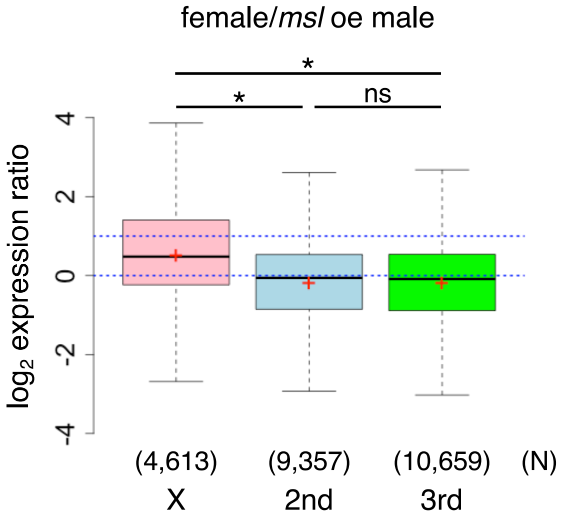

Fig. S8: Log$_2$ expression ratios of transcripts from genes on the X, second, and third chromosomes between female PGCs and male PGCs overexpressing *msl-1, msl-2, msl-3*, and *roX2*.

Log$_2$ expression ratio of the transcripts from genes on the X (pink), second (light blue), and third chromosomes (lime green) between female PGCs and male PGCs overexpressing *msl-1*, *msl-2*, *msl-3*, and *roX2* (female/*msl* oe male) at embryonic stages 15–16. Each box plot represents values as in Fig. 1a. The mean values for the X, second, and third chromosomes were 0.52, –0.17, and –0.17, respectively. Blue dotted lines indicate log$_2$ expression ratios of 0 and 1. Significance was calculated by two-sided Mann–Whitney U test (*, $P < 0.05$; ns, not significant). Transcripts for which TPM was zero in all samples were excluded from this analysis. N: number of transcripts examined. Expression ratio of the transcripts from X-linked genes in female/*msl* oe male was significantly lower than in female/male in Fig. 4a (*P* values, calculated by two-sided Mann–Whitney U test, were < 0.05), and the effect sizes calculated by Cliff's Delta were non-negligible (0.17)[42].

Supplementary Figure S9

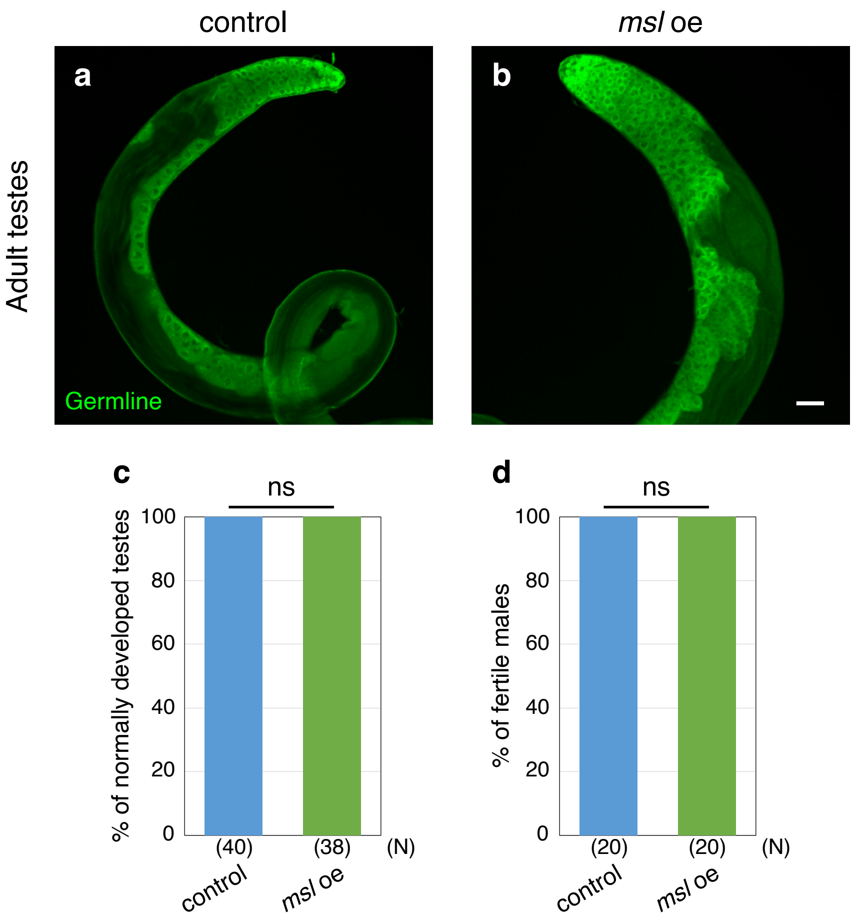

Fig. S9: Phenotypes of flies overexpressing *msl-1*, *msl-2, msl-3*, and *roX2* in germline cells.

(a and b) Testes of progeny derived from *nos-Gal4*/*nos-Gal4* females mated with *y w* males (a, control) and *UAS-msl-3 UAS-roX2*/*UAS-msl-3 UAS-roX2*; *nos-Gal4*/*nos-Gal4* females mated with *UAS-msl-1 UAS-msl-2*/*UAS-msl-1 UAS-msl-2* males (b, *msl* oe). Testes were obtained from adults 3–5 days after eclosion and stained for Vasa (green). Scale bar: 50 µm. **(c)** Percentage of normally developed testes in control (blue) and *msl* oe (green) males. Testes > 1 mm in length were considered to be normally developed. Significance was calculated by two-sided Fisher's exact test; ns, not significant. N: total number of observed testes. We performed two biologically independent experiments and obtained similar results. **(d)** Fertility of control (blue) and *msl* oe (green) males. We separately mated each control and *msl* oe male with *y w* females, and examined whether offspring were produced. Significance was calculated by two-sided Fisher's exact test; ns, not significant. N: number of males examined. Two biologically independent experiments yielded similar results.

Supplementally Table S1: Primers used in this study.

Primer names	Primer sequences (5′–3′)†
msl-1Fw	AGGTCCTGTTCATTG*AAAA*ATGGACAAGCGATTCAAGTG
msl-1Rv	CTGATCCCCGGGCGGCTAACGATTCTTCTGGCGC
msl-2Fw	AGGTCCTGTTCATTG*AAAA*ATGGCCCAGACGGCATAC
msl-2Rv	CTGATCCCCGGGCGGTTACAAGTCATCCGAGCCC
pUASp-K10-attBFw	TATTCTTTTGATTTAAATTGGCCGCTCTAGCCC
pUASp-K10-attBRv	CGGCAAAATCCCTTATCAGCCAATCCGCCGCACCCTC
msl-3Fw	AGGTCCTGTTCATTG*AAAA*ATGACGGAGCTAAGGGACGAG
msl-3Rv	CTGATCCCCGGGCGGCTAAGCAGCAATCCCATCC
roX2Fw	AGGTCCTGTTCATTGATTCGCGGCCTGGTCACA
roX2Rv	CTGATCCCCGGGCGGGACTGGTTAAGGCGCGTAAAAC
msl-1IHFw	GTCTTCGACACAGGCAGAGATAAG
msl-1IHRv	GATAGTCGAAGTGGTGCTGCTAAC
msl-2IHFw	GACTTCAAGACCTACGAGGAGAAC
msl-2IHRv	CTTAAGTGTTGGCTCGTCACTG
msl-3IHFw	GCCTGAGGTTCTACGAGTACAAGA
msl-3IHRv	GTTGTCGTCTGTGAGGTAGTTGTG
mofIHFw	CCTCTTCATCTCAAAGGGACAC
mofIHRv	AGTTGGCAGTATAGTGACTCCTCC
mleIHFw	CTTCGTACTCTAGCACCTTCTCAG
mleIHRv	GCTGTTAGTAAAGCGTCCTGTCTC
roX1IHFw	ATACTGTAGACAAGGAGAGACGGC
roX1IHRv	CTACGCATAACTTTAGGCCAGC
roX2IHFw	TGGCCATCGAAAGGGTAA
roX2IHRv	CACGTCTTTTAAGACTTCAGTTTGC

†Overlapping ends for cloning using the In-fusion HD cloning Kit are underlined. The Kozak sequences for efficient expression of transgenes are indicated in italics.

Supplementally Table S2: The number of PGCs collected for transcriptome analysis.

Biological replicates[†]	No. of male PGCs	No. of female PGCs
1	185,729	186,289
2	218,755	232,502
3	211,261	223,007

[†]PGCs were obtained from three independent batches of embryos.
